# Uncovering Motivational Profiles Among Academically Resilient Students: A Population-Level Latent Profile Analysis

**DOI:** 10.3390/bs16060852

**Published:** 2026-05-26

**Authors:** Michele Zacchilli, Giulia Raimondi, Sara Manganelli, Elisa Cavicchiolo, Tommaso Palombi, James Dawe, Barbara Cazzolli, Fabio Lucidi, Fabio Alivernini

**Affiliations:** 1 Department of Social and Developmental Psychology, University of Rome “La Sapienza”, Via dei Marsi 78, 00185 Rome, Italy; michele.zacchilli@uniroma1.it (M.Z.); sara.manganelli@uniroma1.it (S.M.); barbara.cazzolli@uniroma1.it (B.C.); fabio.lucidi@uniroma1.it (F.L.); fabio.alivernini@uniroma1.it (F.A.); 2 BeSSA Department, Wellbeing, Health and Environmental Sustainability, University of Rome “La Sapienza”, Via delle Fontanelle, 02100 Rieti, Italy; 3 Department of Systems Medicine, Tor Vergata University of Rome, Via Montpellier 1, 00133 Rome, Italy; elisa.cavicchiolo@uniroma2.it; 4 Department of Dynamic and Clinical Psychology and Health Studies, University of Rome “La Sapienza”, Via degli Apuli 1, 00185 Rome, Italy; tommaso.palombi@uniroma1.it; 5 Department of Humanities and Social Sciences, Universitas Mercatorum Telematic University, Piazza Mattei 10, 00186 Rome, Italy; james.dawe@unimercatorum.it; 6 Department of Statistics, Computer Science, Applications (DiSIA), University of Florence, Viale Giovanni Battista Morgagni 59, 50134 Firenze, Italy

**Keywords:** resilient students, motivation, school dropout, latent profile analysis, LPA, Self-Determination Theory, SDT, amotivation, high school students

## Abstract

Academically resilient students achieve high performance despite socioeconomic disadvantages. Although this population has received increasing attention, little is known about its motivational heterogeneity, a critical gap given the central role of motivation in persistence and success. Guided by Self-Determination Theory (SDT), this study examined motivational profiles among a population of academically resilient 10th-grade students in Italy (N = 15,751). Using a person-centered approach, Latent Profile Analysis (LPA) identified three profiles: a “multifaceted regulation resilient” profile (72%), marked by low amotivation and high levels across regulations; a “moderately amotivated resilient” profile (21%), with higher amotivation and lower levels of regulation; and a “strongly amotivated resilient” profile (7%), characterized by the highest amotivation and the lowest levels of regulation. Auxiliary analyses indicated that the amotivated profiles, particularly the “strongly amotivated resilient” profile, exhibited higher school dropout intentions than the “multifaceted regulation resilient” profile. Overall, although the majority of academically resilient students displayed multiple coexisting forms of regulation, a non-negligible subgroup showed significant motivational vulnerability, with amotivation emerging as a central risk factor. These findings challenge the assumption that academic resilience is sufficient to protect students from motivational disengagement and dropout risk. High academic achievement, in other words, should not be taken to imply the absence of motivational concerns. This highlights the importance of moving beyond a one-size-fits-all approach, recognizing that even within resilient populations, specific subgroups remain motivationally vulnerable and in need of tailored support.

## 1. Introduction

Academically resilient students are typically defined as those who are socioeconomically disadvantaged and, nonetheless, achieve high levels of academic performance, outperforming many of their peers (OECD, 2011). Although this definition has been critiqued for relying on a limited set of disadvantage indicators and for the thresholds used to classify students (Alivernini et al., 2016; Ye et al., 2021), it remains the gold standard adopted in large-scale international assessments (OECD, 2023). Given that students with low socioeconomic status (SES) usually perform worse than their peers (Alivernini, 2013; Dietrichson et al., 2017; Kim et al., 2019; Sirin, 2005), resilient students have attracted considerable research attention due to their ability to succeed despite adversity. Motivational variables have emerged as a key factor in this phenomenon (Alivernini et al., 2016; Cui et al., 2023; OECD, 2011; Sandoval-Hernández & Białowolski, 2016; Yan & Gai, 2022). This focus on motivation is particularly important given its well-established role as a protective factor against school dropout (J. L. Howard et al., 2021), a risk to which students from low-SES backgrounds are especially vulnerable (Cavicchiolo et al., 2026).

However, the research on academic motivation with resilient students has relied solely on variable-centered approaches, focusing on average differences between resilient and non-resilient (Gordon Rouse, 2001; Thorsen et al., 2021), which offer limited insight into how motivational patterns vary across individuals (Bureau et al., 2026). As a result, little is known about whether resilient students share similar motivational patterns or whether qualitatively distinct motivational profiles coexist within this group. In this regard, a person-centered approach that focuses on motivational profiles can provide a suitable framework for capturing potential heterogeneity among resilient students and informing more targeted interventions aimed at supporting their long-term academic success.

Bureau et al. (2026), in a recent systematic review of the literature, have noticed that the few research that adopted a person-centered approach within Self-Determination Theory on the general student’s population have consistently identified a few recurring motivational profiles across diverse samples and educational contexts, One commonly observed profile, often labeled “Self-Determined” or “Autonomous”, is characterized by relatively high levels of intrinsic motivation and identified regulation, combined with average to low levels of introjected regulation, external regulation, and amotivation. Another frequently emerging profile, variously labeled “Mixed Motivation”, “Moderately Motivated”, “Conflicted”, or “High Quantity”, displays similar levels across intrinsic motivation and identified, introjected, and external regulations. A third recurring profile, typically labeled “Amotivated”, is defined by very high levels of amotivation alongside average to low levels on the other motivational indicators. However, none of these studies have focused solely on resilient students and this category remains heavily understudied.

Therefore, the aim of the current paper is to investigate whether there are different motivational profiles of resilient students, under the framework of Self-Determination Theory (SDT), and to explore the associations between different profiles and the intention of school dropout, a reliable proxy of actual dropout (Cavicchiolo et al., 2026; Findeisen et al., 2024). In doing so, this study is the first to investigate motivational profiles in a sample of only resilient students.

### 1.1. Academic Motivation of Resilient Students in the Light of Self-Determination Theory

Self-Determination Theory (SDT) conceptualizes academic motivation as qualitatively different reasons why students engage in school-related activities, which vary in the extent to which learning is personally meaningful and internally endorsed (Deci & Ryan, 2000; Ryan & Deci, 2000). Rather than treating motivation as a unitary construct, SDT distinguishes several forms of regulation that can be ordered along a continuum ranging from a lack of motivation to fully self-endorsed motivation (J. L. Howard et al., 2020). At one end of this continuum, amotivation refers to the absence of intention to act, typically associated with low perceived competence and limited value attributed to schoolwork. Moving along the continuum, external regulation describes motivation driven by external demands or consequences, such as grades or sanctions, whereas introjected regulation reflects behavior guided by internal pressures, including feelings of guilt or the need to demonstrate one’s worth. More internalized forms of motivation, further ahead along the continuum, include identified regulation, in which students engage because they recognize the personal importance or usefulness of learning, and intrinsic motivation, characterized by interest and enjoyment in the learning activity itself (J. L. Howard et al., 2017; Ryan & Deci, 2017).

Meta-analytic evidence suggests that different forms of motivation have important and diverse implications for educational outcomes (J. L. Howard et al., 2021): amotivation is robustly associated with maladaptive educational outcomes, including low academic achievement, reduced engagement, lower academic self-efficacy, and stronger dropout-related intentions; external regulation shows weak or nonsignificant associations with achievement and persistence and is often linked to poorer well-being; introjected regulation is more ambivalent: while it is positively related to effort and performance-oriented goals, it is also associated with indicators of ill-being such as anxiety and negative affect; identified regulation shows particularly strong and reliable associations with persistence-related outcomes, including effort, engagement, and lower intentions to drop out of school; finally, intrinsic motivation is consistently associated with higher academic engagement, achievement, and student well-being, reflecting the benefits of learning driven by interest and enjoyment.

Research on resilient students has found that they usually show higher levels of intrinsic motivation and attribute higher value to learning, compared to non-resilient (Cui et al., 2023; OECD, 2011). These findings suggest that resilient students may constitute a subgroup of disadvantaged students at lower risk, as they appear to have developed adaptive ways of endorsing and valuing their education. However, to the best of our knowledge, the only evidence from a person-centered approach, which focused on resilient students, suggested that they might not be a homogeneous group (Jiang et al., 2025). Specifically, distinct motivational profiles characterized by low, moderate, and high levels of both external and intrinsic motivation have been identified among resilient students. However, despite the novel approach, this study focused only on extrinsic and intrinsic motivation and did not provide a comprehensive overview on motivation. Since different forms of motivation can coexist within individuals, focusing on a single or a couple of motivational dimensions may still obscure meaningful heterogeneity (Vansteenkiste et al., 2009). These findings call for analytical approaches that consider motivational patterns at the individual level, thereby providing a strong rationale for the use of person-centered methods in the study of academic resilience.

In contrast to the predominant way to study individual differences in educational settings (i.e., “variable-centered approach”), which estimates “average” relations among variables and implicitly assumes similarity across all students, a “person-centered approach”, aims to identify subgroups of individuals characterized by distinct configurations of personal and educational variables (M. C. Howard & Hoffman, 2018). This approach was recently vastly used to investigate motivational profiles of students, showing its suitability in the educational setting (Bureau et al., 2026). However, no studies have ever investigated whether similar or qualitatively different motivational profiles characterize academically resilient students. The addition of a latent profile approach allows the educational researcher to identify specific types of resilient students that otherwise would remain unseen. Addressing this gap is particularly important because it could enable a more specific and comprehensive understanding of the motivational patterns of resilient students, helping identify potential risk factors and providing evidence to support other disadvantaged students in becoming resilient.

### 1.2. The Present Study

The present study seeks to extend the literature on academically resilient students by adopting a person-centered approach to exploring possible differences within resilient students.

Specifically, the first aim of this study is to investigate whether distinct motivational profiles can be identified within resilient students. To address this aim, a Latent Profile Analysis (LPA; Gibson, 1959; Nylund-Gibson & Choi, 2018) was conducted on the entire population of 10th grade academically resilient students, identifying motivational profiles grounded in Self-Determination Theory. The second aim of the study is to explore the association between these motivational profiles and students’ dropout intentions, to assess whether certain profiles are more strongly associated with educational persistence. To address this second aim, a 3-step approach (Asparouhov & Muthén, 2014; Lanza et al., 2013) was implemented to identify differences between profiles, estimating specific associations with intention to drop out.

## 2. Materials and Methods

### 2.1. Participants

The present study drew on data from the total population of 273,931 10th grade tenth-grade students enrolled in public upper secondary schools in Italy who took part in the National Evaluation of Learning (National Institute for the Evaluation of the Education System, 2015). Consistent with the OECD’s PISA definition, resilient students were selected by identifying those who had a socioeconomic status level in the first quartile of the distribution, and, at the same time, a level of achievement in the fourth quartile of the distribution (OECD, 2023). Following this process, a group of 15,751 resilient students were selected, representing approximately 5.69% of the total student population. The mean age of the students was 15.48 (SD = 0.7) years, and female students accounted for 50.4% of the sample.

### 2.2. Measures

#### 2.2.1. Socioeconomic Status (SES)

The SES of students was determined according to international research (OECD, 2014), such as the Programme for International Student Assessment (PISA), using the index of social, economic and cultural status (ESCS). The ESCS index is derived from four indicators of family background: (i) the highest level of parental education, expressed as years of schooling based on the International Standard Classification of Education (ISCED); (ii) the highest parental occupational status, measured group coding students’ descriptions of their parents’ professions (such as manager, teacher, or clerk) into six tiers ranked by occupational status; (iii) an index of home literacy resources, investigating household items and other resources for learning, such as personal computers or internet access; (iv) the number of books available in the home, in a range from “*0 to 10 books*” to “*more than 500 books*.” After being calculated, each of the indicators was standardized to have a mean of 0 and a standard deviation of 1. The final score was then computed conducting a principal component analysis (PCA) of these indicators. Students were considered socio-economically disadvantaged if they were among the 25% of students with the lowest ESCS.

#### 2.2.2. Achievement

Students’ academic achievement was assessed using scores from the INVALSI national standardized tests of reading literacy and mathematics (INVALSI, 2015). These tests consist primarily of multiple-choice items, with a smaller proportion of open-ended items requiring a single correct response. Each item response is coded dichotomously as correct (1) or incorrect (0). For both mathematics and reading literacy, test items are developed using Item Response Theory (IRT) models. The mathematics test covers several content domains, including algebra, geometry, statistics, and calculation. The reading literacy test includes two main domains: reading comprehension and grammar. Students’ average scores in reading literacy and mathematics were used to identify resilient students as those performing in the top quartile of academic achievement among students in the lowest quartile of SES scores.

#### 2.2.3. Academic Motivation

Academic motivation was measured using the Italian version of the Academic Motivation Scale (AMS; Vallerand & Bissonnette, 1992; Vallerand et al., 1993; Italian version: Alivernini & Lucidi, 2008; Manganelli et al., 2021). Students responded to 20 items assessing their reasons for attending school, indicating the extent to which each statement reflected their personal reasons for attending school. This scale has previously been used in the Italian context, specifically with low-SES students, and has demonstrated satisfactory psychometric properties (Cavicchiolo et al., 2026; Bianchi et al., 2022; Manganelli et al., 2021). The scale measures students’ motivation toward school activities and distinguishes among five types of motivational regulation: amotivation (e.g., “*I don’t know; I can’t understand what I am doing in school*”; α = 0.85), external regulation (e.g., “*In order to obtain a more prestigious job later on*”; α = 0.73), introjected regulation (e.g., “*To show myself that I am an intelligent person*”; α = 0.78), identified regulation (e.g., “*Because eventually it will enable me to enter the job market in a field that I like*”; α = 0.78), and intrinsic motivation (e.g., “*Because I experience pleasure and satisfaction while learning new things*”; α = 0.82). Responses were recorded on a 4-point Likert scale ranging from 1 (“Not at all”) to 4 (“A lot”).

#### 2.2.4. Intention to Drop Out

The intention to drop out of school was measured using a three-item scale that assessed students’ propensity to discontinue their education, as opposed to their intention to persist in school (Hardre & Reeve, 2003; Italian version: Alivernini & Lucidi, 2011; Bianchi et al., 2021; Cavicchiolo et al., 2026). This scale has previously been used in the Italian context, specifically with low-SES students, and has shown satisfactory psychometric properties (Alivernini et al., 2023; Cavicchiolo et al., 2026). Students were asked to indicate how frequently they had considered leaving school (e.g., “*How often do you consider the idea of leaving school*?”) or interrupting their educational pathway (e.g., “*How often do you feel unsure about continuing your studies from one year to the next?*”). Responses were recorded on a 5-point scale ranging from 1 (“Never”) to 5 (“Very often”). In the current study, the Cronbach’s alpha (α) was 0.81.

### 2.3. Analysis

To address the first aim of investigating distinct motivational profiles among academically resilient students, a Latent Profile Analysis (LPA) was conducted using the five forms of academic motivation (amotivation, external regulation, introjected regulation, identified regulation, and intrinsic motivation) as indicators of these profiles. Model estimation and class enumeration followed established recommendations for person-centered analyses (Sorgente et al., 2025; Weller et al., 2020).

For the class enumeration process, several statistical fit indices and classification criteria were considered (Nylund-Gibson & Choi, 2018). These included the Akaike Information Criterion (AIC), the Bayesian Information Criterion (BIC), and the sample-size-adjusted Bayesian Information Criterion (aBIC), with lower values indicating a better fit (Morgan, 2015). Additionally, we also considered the average latent class posterior probability (ALCPP), a value representing the average probability of a person being assigned to a profile given his or her scores on the indicator variables (B. O. Muthén & Muthén, 2000). Values closer to 1 indicate better classification, and a profile is considered acceptably classified when its ALCPP exceeds 0.90. Classification quality was evaluated using entropy, with values of 0.80 or higher indicating adequate separation between profiles (Sorgente et al., 2025). In addition, the adjusted Lo–Mendell–Rubin likelihood ratio test (aLMR) was used to compare models with *k* profiles to models with *k* − 1 profiles, with a statistically significant *p*-value (*p* < 0.05) indicating that the model with an additional profile provides a significantly better fit to the data (Lo et al., 2001).

In accordance with standard guidelines (Sorgente et al., 2025), model estimation began with a one-profile solution and proceeded sequentially by adding one profile at a time. Solutions were compared until: (i) the aLMR test was no longer statistically significant, (ii) information criteria no longer showed meaningful improvement, (iii) entropy remained acceptable, and (iv) the smallest profile included at least 5% of the sample. Once a candidate solution was identified, a Wald test of parameter constraints was conducted to examine whether the levels of the motivational indicators differed significantly across profiles, thereby supporting their substantive distinctiveness.

In relation to the second aim of investigating the associations between the latent class variable and students’ intention to drop out, once the best solution was found, we employed a 3-step approach using intention to drop out as auxiliary distant variable (Asparouhov & Muthén, 2014; Lanza et al., 2013). This procedure accounts for classification uncertainty by first deriving individuals’ most likely class membership based on posterior probabilities and then estimating associations with distal variables, while correcting for classification errors.

All LPA models, Wald tests, and 3-step distal outcome analyses were conducted using Mplus version 8.0 (L. K. Muthén & Muthén, 1998–2017), with 500 random sets of start values and 50 iterations for each random start. The complex sampling structure of the data (students nested within classes) was accounted for using the “COMPLEX” option. Associations with distal outcomes were estimated using the “DU3STEP” procedure. Missing data were handled using full information maximum likelihood (FIML) estimation in Mplus. Descriptive analyses were performed using SPSS version 27 (IBM Corp., 2020; Armonk, NY, USA). Correlations of 0.10, 0.20, and 0.30 were considered indicative of relatively small, typical, and relatively large effects, respectively (Funder & Ozer, 2019; Gignac & Szodorai, 2016).

## 3. Results

Descriptive statistics and Pearson’s correlations of the different forms of regulation and intention to drop out are presented in Table 1. These results align almost perfectly with previous studies supporting the simplex pattern predicted by SDT (J. L. Howard et al., 2020): all regulations were negatively correlated with amotivation and positively correlated with one another, with correlation strength increasing with proximity along the motivational continuum. Regarding the correlations with intentions to drop out, results showed a large negative correlation with amotivation, a small positive correlation with external and introjected regulation, and a medium positive correlation with both identified and intrinsic regulation.

### 3.1. Model Comparison and Selection

The model selection process followed established recommendations for latent profile analysis (Sorgente et al., 2025; Weller et al., 2020). First, we conducted a LPA specifying a one-class solution and progressively increasing the number of classes by one (the results of the fit indices for the various models are presented in Table 2).

The model comparison process ended with the four-class solution, since the Lo-Mendell-Rubin adjusted likelihood ratio tests (aLMR) did not reach significance (*p* = 0.203), suggesting that the four-profile solution does not explain the data better than the three-profile solution. Although the AIC, BIC, and sample-size adjusted BIC continued to decrease with the addition of a fourth profile, such reductions are expected in large samples and were therefore not considered sufficient evidence to support the more complex model (Morgan, 2015; Sorgente et al., 2025; Xie et al., 2020). Moreover, the four-profile solution showed a noticeable decrease in entropy, despite remaining above the 0.80 threshold, suggesting reduced classification precision and the lowest ALCPP for the four-profile solution was below the threshold of 0.90, suggesting classification uncertainty. Taken together, these results support the selection of the three-profile solution as the most parsimonious and interpretable representation of motivational profiles among academically resilient students.

### 3.2. Latent Motivational Profiles

With respect to the first aim, the latent profile analysis revealed that, within the group of academically resilient students, there was substantial heterogeneity in motivational patterns. The three-profile solution provided a clear and parsimonious representation of this differentiation.

Profile 1, which included approximately 72% (n = 11,393) of resilient students, was characterized by lower levels of amotivation compared to the other profiles and by comparatively higher levels of external, introjected, identified, and intrinsic regulation. The latter two forms of regulation, which were slightly higher than introjected and external regulation, showed the greatest differentiation from the other two profiles. We labeled this profile “multifaceted regulation resilient.”

Profile 2, comprising approximately 21% of the sample (n = 3266), displayed a more ambivalent motivational pattern. Compared to the first profile, amotivation was significantly higher, while all the forms of regulation were lower. The differences from Profile 1 were more pronounced for identified and intrinsic regulation, while external and introjected regulations showed more similar levels. Compared to the third profile, Profile 2 exhibited lower levels of amotivation, slightly higher levels of external, identified, and intrinsic regulation, and similar levels of introjected regulation. We labeled this profile “moderately amotivated resilient.”

Profile 3, which accounted for approximately 7% of the sample (n = 1092), presented the most critical motivational pattern. Students in this group reported markedly higher levels of amotivation than those in the other two profiles and lower levels of almost all other forms of regulation. As previously noted, only introjected regulation did not differ significantly from that observed in the second profile. We labeled this profile “strongly amotivated resilient.”

**Figure 1:**
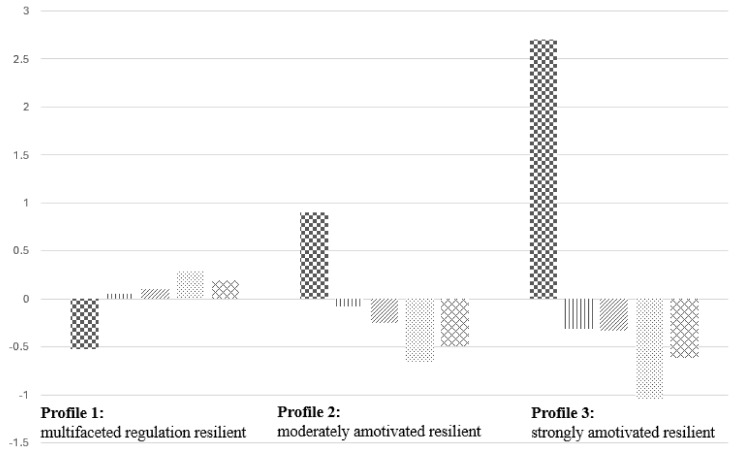
Motivational Profiles of resilient students. Note. The y-axis shows mean standardized (z) scores for each form of regulation.

A Wald-test of parameter constraint showed that the three profiles were, overall, different from each other (Wald χ^2^ = 10,784.944; df = 10; *p* < 0.001), and that the mean values of the regulations were different between profiles, except for introjected regulation between Profiles 2 and 3 (i.e., moderately amotivated and strongly amotivated resilient), whose difference only approached significance (*p* = 0.055). More specifically, amotivation was highest in Profile 3 (i.e., strongly amotivated resilient), followed by Profile 2 (i.e., moderately amotivated resilient), and lowest in Profile 1 (i.e., multifaceted regulation resilient). A similar ordered pattern emerged for all other forms of motivation. For external regulation, introjected regulation, identified regulation, and intrinsic motivation, the lowest levels were observed in Profile 3 (i.e., strongly amotivated resilient), intermediate levels in Profile 2 (i.e., moderately amotivated resilient), and the highest levels in Profile 1 (i.e., multifaceted regulation resilient). For ease of visualization, the mean standardized scores (z-scores) of the final three-profile solution are illustrated in Figure 1.

Additionally, Table 3 reports the raw means and standard deviations of the five clustering variables, together with the number of students in each profile, and a summary of the differences in regulations between profiles is shown in Table 4.

In relation to the second aim, results from the 3-step analysis indicated significant differences among the three motivational profiles in students’ intentions to drop out of school (overall test: χ^2^ = 3378.25, *p* < 0.001). Profile 1 (i.e., multifaceted regulation resilient) exhibited the lowest level of dropout intention (M = 1.56, SE = 0.01), followed by Profile 2 (i.e., moderately amotivated resilient; M = 2.61, SE = 0.02) and Profile 3 (i.e., strongly amotivated resilient; M = 3.04, SE = 0.04). Pairwise comparisons using Tukey’s HSD revealed significant differences between all three profiles (all *p* < 0.001), with effect sizes ranging from large to very large (Hedges, 2025): as stated before, Profile 1 reported lower dropout intentions than both Profile 2 (d = 0.91) and Profile 3 (d = 1.64), and Profile 2 reported lower dropout intentions than Profile 3 (d = 0.73).

## 4. Discussion

The purpose of this study was to investigate motivational profiles of resilient students, defined as those who, despite low socioeconomic status, can reach high levels of performance at school, and to explore the association between latent profile membership and intention to drop out of school. By identifying qualitatively different motivational configurations, the present study provides a more nuanced understanding of the motivational mechanisms that may sustain resilience, as well as inform the development of more targeted educational interventions.

Before discussing the LPA results, we briefly address the bivariate correlations, as the observed relationships between intention to drop out and motivation diverge from those reported in the general student population in previous studies. The literature has consistently reported positive correlations between dropout intentions and controlled motivation, and negative correlations with autonomous motivation (Rump et al., 2017; Jeno et al., 2018; Jeno et al., 2023; Dagani et al., 2026). Conversely, the present study observed negative correlations with all forms of regulation, suggesting that in academically resilient students, both controlled and autonomous regulations are associated with lower intentions to drop out. These unexpected findings are consistent with the broader pattern of results observed in this study and suggest the possibility that resilient students may draw upon any form of regulation to sustain their academic motivation, regardless of its position on the SDT continuum. This hypothesis will be further explored in the following sections, where the LPA results are discussed.

### 4.1. The Motivational Profile of Resilient Students

The investigation of motivational profiles among resilient students revealed three distinct profiles that differed in both prevalence and motivational configuration. Students in the first profile (i.e., *multifaceted regulation resilient*), which represented around three-quarters of all resilient students, reported relatively higher levels of identified and intrinsic regulation compared to students in the other profiles, suggesting that they can sustain high academic performance not only because of their academic abilities, but also because they attribute personal meaning to learning and derive enjoyment and satisfaction from it. At the same time, this profile also exhibited higher levels of external and introjected regulation, indicating a greater influence of both external contingencies and internalized pressures relative to the other profiles. With respect to amotivation, students in this group reported lower levels, suggesting that the majority of all resilient students have a clear sense of purpose regarding school attendance. Taken together, this profile did not exhibit a pronounced orientation toward either extreme of the self-determination continuum but rather reflects a complex motivational configuration in which autonomous and controlled forms of regulation coexist. This profile was also associated with lower dropout intentions than the other profiles, suggesting that most resilient students may be better positioned for future academic success.

The second profile (i.e., *moderately amotivated resilient*), comprising approximately 21% of the sample, was characterized by higher levels of amotivation and lower levels of all forms of regulation compared to the multifaceted regulation profile. External and introjected regulation, although remaining below the mean for the full resilient sample, were the highest forms of regulation of this profile, suggesting that motivation for school is primarily guided by internalized and external pressures. Regarding outcomes, the students in this profile were found to have significantly higher intentions to drop out than those in the multifaceted regulation profile. This pattern is consistent with a potential risk condition: although these students maintain high academic achievement, their elevated dropout intentions and low levels of autonomous regulation represent a substantial risk for long-term educational persistence.

The third profile (i.e., strongly amotivated resilient), which accounted for approximately 7% of the sample, presented the most critical motivational pattern. Students in this group reported markedly higher levels of amotivation and lower levels of nearly all forms of regulation, compared to the other profiles, with the lowest levels observed in identified regulation. Introjected regulation, however, did not differ significantly from that observed in the second profile, suggesting that even in the presence of higher amotivation, students with this profile still experience some degree of internal pressure (e.g., feelings of obligation or guilt). In other words, although they mostly lack self-determination in their actions, they may still be driven to some extent by self-imposed expectations.

Given that introjected regulation has been associated with increased anxiety (J. L. Howard et al., 2021), the well-being of students in this profile may be particularly at risk. Moreover, their motivational vulnerability is further underscored by the outcome measures, as this group was associated with the highest levels of intention to drop out.

### 4.2. Theoretical Implications for Academic Resilience

These results invite reflection on the interpretation of academic resilience as a positive characteristic altogether, considering that it can coexist with motivational difficulties. Resilience, for students with profile 3, might involve hidden psychological costs, raising questions about its long-term sustainability and, considering the negative effect of amotivation on performance (J. L. Howard et al., 2021), its implications for future students’ success.

These results contribute to the ongoing debate within motivational theories regarding the relative importance of the quality versus the quantity of motivation (Deci & Ryan, 2000; Vansteenkiste et al., 2009). Previous research on motivational profiles in the general student population has consistently shown that the most academically adaptive profile is typically characterized by predominantly autonomous forms of motivation, supporting the notion that the importance of the quality of motivation may matter mote that its quantity (Ratelle et al., 2007; Šakan et al., 2024; Tóth-Király et al., 2022; Wang et al., 2017; Xie et al., 2020). Nevertheless, studies also indicate that, following the autonomously motivated profile, a second adaptive profile often emerges, characterized by high levels of both autonomous and controlled regulations. This profile frequently outperforms profiles marked by exclusively controlled motivation or by uniformly low motivation, suggesting that the overall levels of different types of motivation may still play a meaningful role. In this regard, the present findings are noteworthy: within a sample of low–socioeconomic status high-achieving students, no profile characterized by predominantly high autonomous motivation was identified. Instead, the students of the most predominant and adaptive profile exhibited a motivational pattern marked by moderate levels across all forms of regulation. This pattern suggests that although structural disadvantages associated with low socioeconomic status may constrain the development of strong autonomous motivation, academically resilient students may nevertheless sustain high achievement by mobilizing an integrated combination of motivational regulations, rather than adopting a motivational structure with generally elevated multiple types of regulations. Such findings highlight the potential adaptive value of motivational coexistence under conditions of structural constraint. However, more studies are needed to support this claim.

It is essential to emphasize that the differences between profiles cannot be explained in terms of ability or academic performance, as all students included in the analysis are among the top performers. This suggests that academic resilience may mask very different motivational experiences: some students attend school because they perceive it as meaningful and aligned with their goals, others because they recognize its instrumental value, and still others because they feel obliged to continue, despite not attributing genuine value to their studies. From this perspective, amotivation emerges as a key interpretive construct for understanding why equally resilient students can differ so markedly in terms of dropout intention.

Overall, these results illustrate that academic resilience can conceal qualitatively different forms at the motivational level. While the majority of resilient students also appear to have coexisting forms of regulation, a non-negligible share shows signs of vulnerability that may be linked to a higher risk of disengagement or school dropout despite strong academic performance. This distinction has important theoretical and practical implications, underscoring the need for differentiated interventions that go beyond supporting achievement alone and, instead, also promote more self-determined and sustainable forms of motivation in all students, even those who, apparently, do not perceive the disadvantages associated with low socioeconomic status (Alivernini et al., 2023).

### 4.3. Implications for Educational Research and Practice

These findings have important implications for both educational research and educational practice. From a research perspective, the identification of distinct motivational profiles among academically resilient students challenges the traditional concept of resilience as a uniform construct defined solely by high achievement under disadvantage (OECD, 2011). The results highlight the value of person-centered approaches for uncovering heterogeneity that remains hidden in variable-centered analyses and suggest that future resilience research should systematically incorporate motivational processes, particularly amotivation, as central explanatory dimensions rather than correlates.

Notably, although students in the strongly amotivated resilient profile represent a minority of resilient students, they nevertheless account for more than one thousand adolescents whose academic persistence is at substantial risk. Given both their specific motivational characteristics and their non-negligible size, this group warrants particular attention, particularly because they are at an elevated risk of school dropout. Failure to provide adequate support for these students raises important concerns, as they demonstrate academic capability yet may still be at heightened risk of discontinuing their educational trajectories. Furthermore, these results may underscore the need to move beyond achievement-based definitions of success and to integrate indicators of motivational quality when studying educational equity and long-term outcomes. From a practical standpoint, the findings suggest that high academic performance among socioeconomically disadvantaged students should not be interpreted as an unequivocal signal of low risk. While most resilient students show a more prevalent mixed motivation profile, a substantial minority still exhibit motivational vulnerabilities that may compromise their educational persistence over time. Educational interventions should therefore be differentiated and tailored to students’ motivational profiles rather than uniformly targeted at “resilient” students as a homogeneous group (Azevedo et al., 2023; Tan, 2024). In particular, students characterized by “moderately amotivated” or “strongly amotivated” profiles may benefit from interventions aimed at reducing amotivation and fostering a stronger sense of meaning and autonomy in learning, for example, through autonomy-supportive teaching practices (Banerjee & Halder, 2021; Cheon & Reeve, 2015). Importantly, these results suggest that promoting educational equity cannot be achieved only by supporting the academic achievement of low-SES students, but also by cultivating more self-determined and sustainable forms of motivation that can foster long-lasting academic success, even among students who outwardly appear to be already successful.

### 4.4. Limitations

Although this study analyzed the entire population of 10th grade resilient students in Italy, several limitations must be acknowledged. First, we relied on self-reported measures of motivation and intention to drop out which, although they have been widely used in educational research, may still have introduced self-report bias (Pekrun, 2020). Second, the National Evaluation of Learning is a cross-sectional study, which limited the analysis to a single time point, which precludes strong inferences regarding the temporal relationships among the variables of interest. This is an important limitation, even though intention to leave school is a good predictor of actual school dropout (Cavicchiolo et al., 2026; Findeisen et al., 2024). Third, when we measured the relationship between the latent profile variable and the distal variables, although a three-step approach was used to account for classification error, profile assignment remains probabilistic and may still introduce uncertainty in the estimation of associations with distal outcomes (Asparouhov & Muthén, 2014). Fourth, the data derives from the 2015 national assessment. Although the SDT framework posits that the psychological processes underlying motivational regulation are relatively stable across historical contexts (Ryan & Deci, 2020; Ryan et al., 2025), significant societal changes (e.g., the COVID-19 pandemic) may have altered students’ motivational profiles in ways that are not captured by the present data. Readers should therefore exercise caution when generalizing these findings to the current Italian school context.

### 4.5. Future Directions

Several avenues for future research emerge from the present findings. First, longitudinal designs are needed to examine the stability and developmental trajectories of motivational profiles among academically resilient students, as well as transitions between profiles over time. Such designs would allow researchers not only to determine whether students characterized by “moderately amotivated” or “strongly amotivated” profiles remain resilient in the long term, but also whether motivational vulnerability predicts later disengagement, declines in achievement, or school dropout. Second, future studies should investigate the contextual and educational factors that contribute to the emergence of different motivational profiles, particularly in socioeconomically disadvantaged contexts. Third, since the data derives from a national assessment of 2015, future studies should explore whether great societal changes have influenced motivational profiles of resilient students. Finally, intervention-oriented research is needed to test whether targeted, profile-sensitive approaches, especially those aimed at reducing amotivation and strengthening identified and intrinsic regulation, can promote more adaptive forms of academic motivation and improve persistence among disadvantaged high-achieving students. Together, these directions would advance a more dynamic and context-sensitive understanding of academic resilience and inform policies and practices aimed at supporting long-term educational success.

## 5. Conclusions

The present study contributes to advancing the literature on academic resilience by providing evidence that high academic achievement among socioeconomically disadvantaged students coexists with qualitatively different motivational configurations. Using a latent profile analysis grounded in Self-Determination Theory, this research suggested that, although the majority of resilient students (i.e., Profile 1, *multifaceted regulation resilient*) exhibit a motivational pattern characterized by low amotivation, moderate levels of all forms of regulation, and low intentions to drop out, a substantial minority does not share this adaptive profile. Specifically, more than one quarter of resilient students display motivational patterns characterized by elevated dropout intentions, indicating increased vulnerability with respect to educational persistence and future academic trajectories. This vulnerable subgroup can be further differentiated into two profiles. A larger group, representing approximately 21% of resilient students (i.e., Profile 2, *moderately amotivated resilient*), is characterized by higher levels of amotivation and lower levels of all forms of regulation compared to the majority profile. A smaller group (i.e., Profile 3, *strongly amotivated resilient*) emerges as particularly at risk, as its markedly high levels of amotivation and dropout intention, relative to both Profiles 1 and 2, point to an elevated risk of school disengagement despite strong academic performance.

Taken together, these findings underscore that academic resilience is more complex than previously conceptualized and that achievement-based indicators are not suited to capture this heterogeneity. Recognizing and addressing this heterogeneity is essential for advancing theoretical models of resilience and for designing educational policies and interventions that support not only academic performance, but also the motivational profile necessary for long-term educational success.

## Figures and Tables

**Table 1:** Means, Standard Deviation, and Pearson’s correlations of the variables measured.

Variables	Mean	SD	1.	2.	3.	4.	5.	6.
1. Amotivation	1.46	0.64	1					
2. External Regulation	3.08	0.63	−0.08	1				
3. Introjected Regulation	2.50	0.70	−0.14	0.33	1			
4. Identified Regulation	3.21	0.63	−0.45	0.30	0.36	1		
5. Intrinsic Regulation	2.81	0.65	−0.30	0.03	0.39	0.48	1	
6. Intention to drop out	1.90	0.93	0.49	−0.06	−0.13	−0.29	−0.26	1

Note. SD = Standard deviations; All correlations are statistically significant (*p* < 0.01).

**Table 2:** Comparison of fit indices and likelihood ratio tests for latent profile analysis.

No. of Profile Groups	No. of Free Parameters	Log Likelihood	AIC	BIC	aBIC	aLMR	Entropy	Sgf	ALCPP
1	10	−77,855.364	155,730.729	155,807.375	155,775.596			100%	1
2	16	−72,434.991	144,901.982	145,024.616	144,973.769	0.00	0.882	17.8%	0.92
3	22	−70,161.744	140,367.488	140,536.110	140,466.196	0.00	0.922	6.9%	0.93
4	28	−68,611.994	137,679.988	137,894.599	137,805.617	0.20	0.848	6.5%	0.77

Notes. AIC = Akaike’s Information Criterion; BIC = Bayesian Information Criterion; aBIC = Sample Size Adjusted BIC; aLMR = adjusted Lo-Mendell-Rubin Adjusted Likelihood Ratio Test; Sgf = Smallest group frequency; ALCPP = average latent class posterior probability.

**Table 3:** Number of students, means and standard deviations of the regulations in each profile group.

	Profile 1	Profile 2	Profile 3
Number of students per profile group	11,393	3266	1092
Percentage of all students per profile group	72%	21%	7%
Amotivation	1.13 (0.20)	2.04 (0.27)	3.19 (0.43)
External Regulation	3.11 (0.61)	3.03 (0.66)	2.88 (0.74)
Introjected Regulation	2.58 (0.68)	2.33 (0.67)	2.27 (0.85)
Identified Regulation	3.39 (0.51)	2.79 (0.62)	2.55 (0.75)
Intrinsic Regulation	2.94 (0.60)	2.49 (0.61)	2.41 (0.82)

Note. Standard deviations are presented in parentheses; Profile 1 = “Multifaceted Regulation Resilient”; Profile 2 = “Moderately Amotivated Resilient”; Profile 3 = “Strongly Amotivated Resilient”.

**Table 4:** Differences in regulations between profiles.

Dimension	Profile Ordering	Note
Amotivation	P3 > P2 > P1	All pairwise profile differences are statistically significant.
External regulation	P1 > P2 > P3	All pairwise profile differences are statistically significant.
Introjected regulation	P1 > P2 and P1 > P3; P2 ≈ P3	P2 vs. P3 difference is not statistically significant (*p* = 0.055).
Identified regulation	P1 > P2 > P3	All pairwise profile differences are statistically significant.
Intrinsic regulation	P1 > P2 > P3	All pairwise profile differences are statistically significant.

Note. Profile 1 = “Multifaceted Regulation Resilient”; Profile 2 = “Moderately Amotivated Resilient”; Profile 3 = “Strongly Amotivated Resilient”.

## Data Availability

Publicly available datasets were analyzed in this study. This data can be found here upon free registration: INVALSI—Statistical Office https://serviziostatistico.invalsi.it/catalogo-dati (accessed on 24 September 2024).
